# An In-Depth Review of the Azygos Vein and Its Clinical Significance

**DOI:** 10.3390/biomedicines13051013

**Published:** 2025-04-22

**Authors:** Alexander Stolarczyk, Nazar Włodarczyk, Nicol Zielinska, Kacper Ruzik, George Triantafyllou, Maria Piagkou, Łukasz Olewnik

**Affiliations:** 1 Department of Anatomical Dissection and Donation, Medical University of Lodz, 90647 Lodz, Poland; alexander.stolaczyk@stud.umed.lodz.pl (A.S.); nazuri91@gmail.com (N.W.); 2 Department of Clinical Anatomy, Mazovian Academy in Plock, 90402 Płock, Poland; n.zielinska@mazowiecka.edu.pl (N.Z.); kacper.ruzik@umed.lodz.pl (K.R.); 3 Department of Anatomy, School of Medicine, Faculty of Health Sciences, National and Kapodistrian University of Athens, 11527 Athens, Greece; georgerose406@gmail.com (G.T.); piagkoumara@gmail.com (M.P.)

**Keywords:** azygos vein, variation, hemiazygos vein, accessory hemiazygos vein

## Abstract

The azygos vein is a vessel that drains deoxygenated blood from the posterior wall of the thorax and upper abdomen into the superior vena cava. It can vary morphologically in both adults and fetuses in origin, termination, diameter, course, and tributaries, especially the hemiazygos vein and accessory hemiazygos vein, which, together with the azygos vein, form the azygos venous system. The main aim of this review is to summarize the current state of knowledge regarding the azygos vein. Another aim is to present the morphological variations of the azygos vein and their clinical significance. This information may be useful to clinicians, especially surgeons. This review also provides an overview of the embryological development of the azygos venous system and the consequences of its developmental errors, as well as their relationship to certain clinical cases and pathologies.

## 1. Introduction

The azygos vein (AV) is one of the primary veins within the azygos venous system (AVS). This unpaired vein is formed through the union of the right subcostal veins and the right ascending lumbar vein at the level of the 12th thoracic vertebra (T12). Along its course, the AV follows a path behind the right crus of the diaphragm, passes through the aortic hiatus, and enters the thorax, where it curves around the base of the right lung before joining the superior vena cava (SVC). Ordinarily, it courses along the right side of the vertebral column (VC). The AV receives deoxygenated blood from the posterior walls of the thorax and upper abdomen, which it then transports to the SVC. Its main tributaries are the hemiazygos vein (HAV), the accessory hemiazygos vein (AHAV), and the 2nd–12th right intercostal veins (ICVs). Due to multiple anastomoses with the inferior vena cava (IVC), the AV connects the IVC and the SVC [1]. The AV has many morphological variants. In most cases, the AV is the AVS’s dominant vein; in others, it seems to be roughly the same size as one of its tributaries, the HAV. In some of the researched cadavers, AV and HAV even had the same origin point. The clinical significance of AV is very much worth noting, as it allows proper blood return from the inferior part of the body to the right atrium in case of an existing blockage in the IVC. Because of the course of the AV in the thorax, in CT scans of the right lung, the AV can be mistaken for a lung pathology [2].

This research presents data on variations in the origin, termination, diameter, course, and tributaries of the AV in both adults and fetuses. To comprehend the genesis of this variability, we detailed the AV’s embryological and evolutionary development. The primary objective of this review is to summarize the available data literature on the AV morphological variants and provide information on their clinical significance.

## 2. Embryology

**Figure 1:**
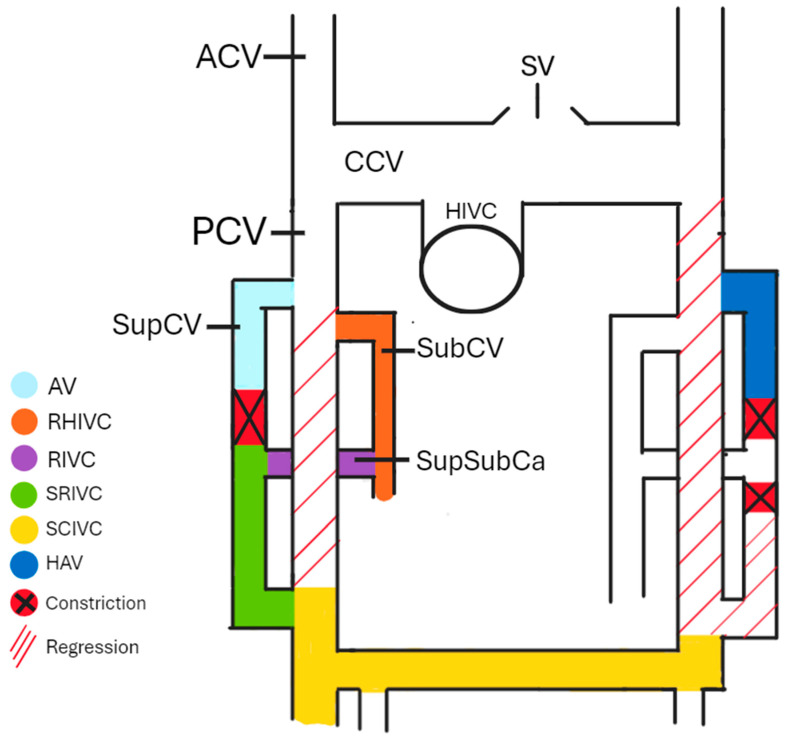
The cardinal venous system. AV—azygos vein, RHIVC—reno-hepatic inferior vena cava, renal inferior vena cava—RIVC, sacrorenal inferior vena cava—SRIVC, sacrocardinal inferior vena cava—SCIVC, HAV—hemiazygos vein, ACV—anterior cardinal vein, PCV—posterior cardinal vein, CCV—common cardinal vein, HIVC—hepatic inferior vena cava, SubCV—subcardinal vein, SupSubCa—supracardinal and subcardinal anastomosis, and SV—superior vena cava.

Understanding the embryogenesis of AV can help us comprehend the origin of many morphological variations. The cardinal venous system is formed by the anterior cardinal veins (ACVs), common cardinal veins (CCVs), posterior cardinal veins (PCVs), subcardinal veins (SubCVs), and supracardinal veins (SupCVs). During the embryo’s development, some anastomoses between particular cardinal veins (CVs) constrict and degenerate, forming a part of the mature venous system. Around the 4th and 5th weeks of the embryo’s development, SupCVs and SubCVs begin to arise from the proximal parts of the PCVs on both sides of the body. On the right side of the body, the right PCV almost completely regresses after the 5th week of development [3], apart from a small proximal part that forms the azygos arch and a short caudal part that forms a part of the IVC. The right SupCV then constricts superiorly to the suprasubcardinal anastomosis (SupSubCa), leading to the right SupCV’s separation. The proximal remnant of the post-constriction-right SupCV forms the AV, while the distal part forms the sacrorenal IVC (SRIVC). The left PCV completely regresses, leading to the left SupCV losing its anastomosis with the left ACV. If this anastomosis remains patent, the HAV in the later stages of development drains into the left brachiocephalic vein (BCV). The left ACV and the interanterior cardinal anastomoses form the left BCV. The involuted left CCV remains as the oblique vein of the left atrium. The left SupCV constricts superiorly and inferiorly to the left SupSubCa, leading to a regression of the distal part of the left SupCV. A left-sided IVC is formed if the inferior part of the right SupCV regresses instead of the inferior part of the left SupCV. Duplicated IVC is present in cases where both SupCVs stay patent at their distal parts. The IVC derives from the right part of the cardinal venous system, apart from a small hepatic part (HIVC) originating from the vitelline veins. As shown in Figure 1, SubCV, SupSubCa, SupCV, and both of the PCVs, respectively, give existence to the reno-hepatic (RHIVC), renal (RIVC), sacrorenal (SRIVC), and sacrocardinal (SCIVC) parts of the IVC [4]. If a connection between the RHIVC and the HIVC fails to develop correctly, the IVC drains directly into the arch of the AV. In that case, the hepatic veins drain independently into the right atrium. Both SupCVs are connected by an anastomosis that, together with the superior part of the left SupCV, is later a part of the HAV [5,6,7].

## 3. Evolution

Galen of Pergamon originally described the AV. The AV is so named because it lacks a counterpart on the left side of the body; “azygos” translates to “without a pair” in Greek [8]. The most primitive version of an AVS is one with a right and a left AV, with the left AV being longer, larger in diameter, and having more tributaries than the right one. In such cases, the right AV drained into the SVC, and the left AV, in most cases, drained directly into the right atrium. In Lemur albifrons, a more advanced version of the AVS was found, with the HAV arising from the inferior part of the AV. The HAV later ran on the left side of the VC and received blood from the left ICVs. In higher primates, there is only one AV, the right one. In some, a HAV can also be found [9,10].

## 4. Morphological Variants of the AV in Adults

The AV has many variants that can be erroneously interpreted as pathological conditions; thus, knowing its different possible routes is crucial. The AV differs in origin, placement along the VC, diameter, connections with the HAV and the AHAV, tributaries, and termination point [1].

## 5. Origin of the AV

Bergmann [1] described that the AV arises from the connection of a combination of lateral, intermediate, and medial roots at the 12th thoracic vertebra (T12) level. The ascending and subcostal veins form the lateral root. The intermediate root rises from the dorsal side of the IVC at the level of the 2nd lumbar segmental vein. The medial root is created by a plexiform complex associated with the ventral side of the lumbar vertebrae and their segmental branches [11].

Pirya et al. [12], in their study of 30 human cadavers, found that in 56.7% of the cases, the AV was formed by a single root; in 36.7%, it was formed by the lateral and intermediate root, and in 6.6% of the cases, it was formed by the union of the lateral and medial root.

An abnormal AV and HAV origin were described in a case study performed by Koutsouflianiotis et al. [13], which found both of these veins originating from a single branch of the IVC. This single venous branch was then divided into AV and the HAV at the level of L1/L2.

The AV can also directly branch off of the IVC. In a case study performed by Lachkar et al. [14], the AV originated from the IVC at the L2 level, then split into two veins at the L1/T12 level that had transvertebral connections between each other at the T9 and T7 levels. The right trunk of this venous plexus drained into the SVC, while the left trunk drained the left ICVs. The HAV and the AHAV were absent in this case.

## 6. Connection with HAV and AHAV

Anson and McVay conducted a study describing AV’s different course patterns and termination points. The results were classified into the following three types:

*Type 1:* Two parallel veins on both sides of the VC, with no connections.

*Type 2*: The most typical type of AVS, with a separate AV, HAV, and AHAV. The AV and the HAV had multiple retroaortic anastomoses between them.

*Type 3:* A single AV lying on the midline on the ventral side of the VC [11].

**Figure 2:**
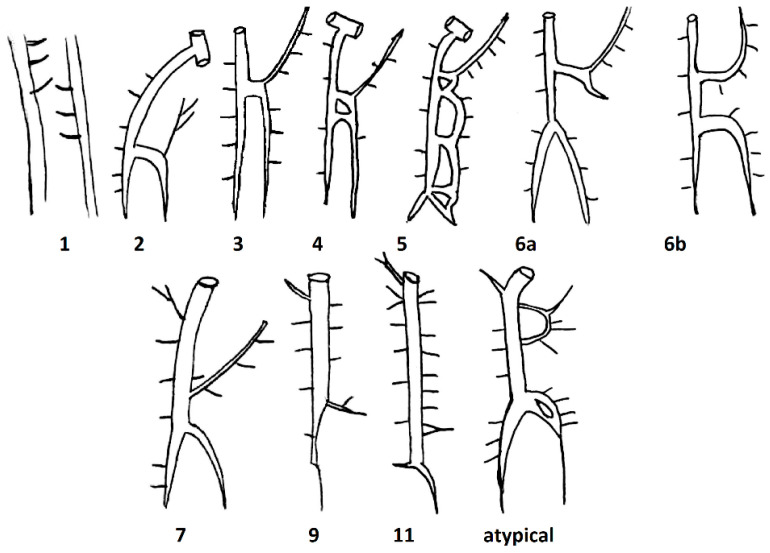
Schematic drawings of the different subtypes found by Kutgolu et al.

Based on the study of Anson and McVay, Kutoglu et al. further divided these three main types, which were initially described by Anson and McVay, into 11 subtypes by dissecting 48 cadavers. *Subtype 1* corresponded to *Type 1*, *subtypes 2–10* corresponded to Type 2, and *subtype 11* corresponded to *Type 3* (Table 1, Figure 2).

The authors found no cases corresponding to groups 8 and 10. In addition to the 11 groups mentioned, they also found an atypical case. The HAV of the atypical case is divided into two veins, which immediately after being connected begin to drain into the AV as a single structure. The AHAV in this case had an anastomosis with one of the ICVs [15].

Ozdemir et al. [16] described an atypical type of AVS in their case study. In their case, there was a single AV to which the 8th–11th ICVs drained directly. The 4th–7th ICVs formed two superior and inferior trunks that drained into the AV. The superior trunk obliquely passed the VC between the aorta and the esophagus and opened into the AV at the T4 level. The inferior trunk passed posteriorly to the aorta.

## 7. Termination of the AV

The AV typically arches around the right main bronchus and drains into the SVC; however, there have been cases of the AV losing touch with the root of the right lung. In that case, the AV grooves into the right lung, creating an azygos lobe. The fissure in which the AV lies is called the azygos fissure, and it separates the azygos lobe from the superior lobe [1].

In their review, Tatar et al. [17] described the levels of the AV arch based on 200 CT angiographies, with the T5 level being the most common (55%) and the T6 level being the rarest (5%). The AV arch at the T4 level was present in 40% of the subjects.

In their study, Kutoglu et al. [15] presented the termination levels of the AV (connection point of the AV and the SVC). The most common termination level was at the T3 vertebral level (66.7%). Other termination levels were the level of a vertebral disc between vertebrae T3 and T4 (6.7%), the T2 vertebrae level (12.5%), and the level of the vertebral disc between T2 and T3 vertebrae (12.5%) (Table 2).

Koutsouflianiotis et al. [18] reviewed CT angiographies of 25 male and 26 female patients. In their study, they found that T5 was the most common termination point of the AV (56.9%). Less common termination points were T4 (31.4%), T6 (9.8%), and T3 (2%).

Priya et al. [12] noted that the AV terminated at the level of the T4 vertebra in 70% of the cases, at the T3 level in 20%, and at the T5 level in 10%.

In their cadaveric study, based on 20 cadavers from the Eastern Indian population, Mohanty et al. [19] found that the AV terminated at the T3 level in 55% of the cases, at the T3–4 level in 20%, T2–3 in 15% and at T4 and T2 both in 5%.

A morphological variant between the AV and left SVC was described by Li et al. [20]. The cadaver had a right and left SVC in the case they described. The AV was formed by both left and right ascending lumbar veins and ascended on the left side of the VC. The 5th–11th ICVs drained directly into the single AV. At its termination point, the AV created an arch around the aorta at about the level of connection between the aortic arch and the descending aorta and drained into the left SVC.

## 8. Tributaries

As the main vein of the AVS, the AV receives blood from the HAV and the AHAV. On top of that, the AV gets blood from the supreme ICVs and multiple intercostal and lumbar veins. Sometimes, the AV can receive blood from the pulmonary, right internal thoracic, superior phrenic, bronchial, and gonadal veins [1].

## 9. Diameter of the AV

The AV may also vary in diameter. Determining the average physiological diameter of the AV can be important in diagnosing and treating conditions affecting this vein, such as aneurysms or indentations caused by malignant processes [17].

Koutsouflianiotis et al. [21] presented a study investigating, among other things, the diameter of the AV. Their methods were based on computed tomography of 25 male and 26 female Greek patients with a mean age of 66.5 years. The results showed that the mean diameter was 0.96 cm. The smallest AV was 0.59 cm, and the largest was 1.58 cm. There was a gender dimorphism with male predominance. The mean values were 1.02 cm for males and 0.91 cm for females. The researchers also found a slight correlation with age. The AV diameter was higher in older patients (1st 0.86 cm and 2nd 0.99 cm).

Tatar et al. [17] carried out another study. They used chest CTs of 103 Turkish patients, 42 females and 61 males. The group was selected from people with no known chest pathology and aged 18 to 83 (median: 47 years). The sex distribution of the subjects is shown in Table 3. 

The results were as follows. The AV diameter at the entrance to the SVC ranged from 4.3 mm to 16 mm. The overall mean diameter was 8.1 mm (7.8 in females and 8.2 in males). No correlation was found between age and AV diameter. Table 4 summarizes the results [17].

Kutoglu et al. [15] published a study of 48 conserved human cadavers aged 27 to 70 years (mean age 48.29), of which 13 were female and 35 were male. The researchers measured the diameter of the AV at its origin and termination level. The mean diameter of AV at its origin was 4.05 mm, ranging from 2.0 to 7.5 mm, and at its termination was 8.56 mm, ranging from 5.0 to 12.2 mm. Table 5 below shows the results of this study.

## 10. Course of the AV

**Figure 3:**
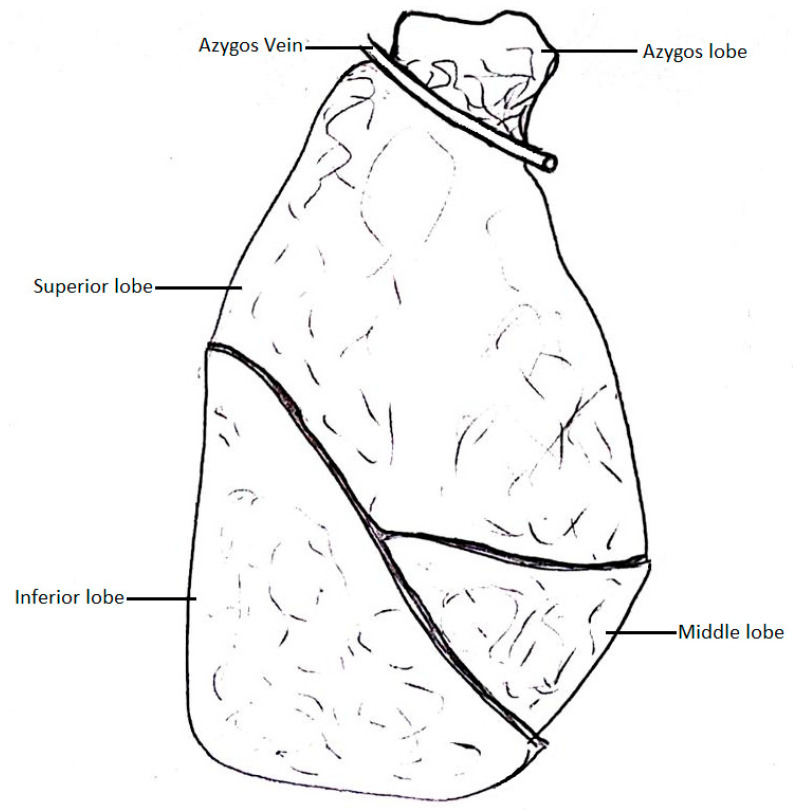
Pulmonary lobe of the azygos vein.

Anson et al. [22] conducted a meta-analysis to define the incidence of the azygos lobe of the right lung (Figure 3). This variation is caused by the azygos arch losing contact with the superior surface of the right main bronchus and grooving the right lung with the base of an accessory pleural fissure before entering the posterior wall of the SVC.

**Figure 4:**
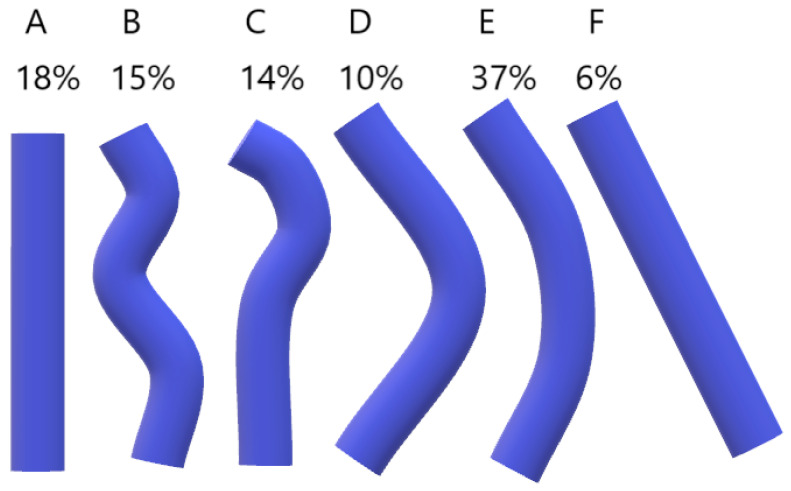
Categories of the Azygos vein (AV) course. (A, straight; B, sinuous; C, hooked; D, elbow; E, bowed; F, diagonal).

In *Bergman’s Comprehensive Encyclopedia of Anatomic Variations*, it is mentioned that the AV can rarely pass through the aortic hiatus of the diaphragm and open directly into the right atrium, which is attributed to the disappearance of the right common cardinal vein during embryonic development [1]. Bales [23] studied the course of the AV in 84 embalmed cadavers with removed viscera overlying the thoracic vertebrae between the aortic hiatus and tracheal bifurcation. Measurements were collected using a scalable grid overlay on a digital photograph of the internal midline view of the VC. The course of the AV was divided into five levels. Superiormost—around T4, Superior intermediate, Middle, Inferior intermediate, and Inferiormost—around T12. The horizontal position of the AV was measured at each level and classified into seven types. The results are presented in Table 6. Bales also suggested different types of overall shape for the AV (Figure 4). The most common shape of the AV was the bowed shape (E).

For this review, we examined several described and published case studies.

Paik [24] described a case in which the AV arises from the level of the T12 body, ascends longitudinally in the right extradural space to the level of the T4 body, then passes through the T4-T5 right intervertebral foramen, courses anteriorly along the right lateral surface of the T4 body, curves just above the right main bronchus to enter the posterior aspect of the SVC 1.7 cm above its entry into the right atrium. Another rare phenomenon is the absence of the AV, which has only been reported in a few patients [25]. Keskin et al. [26] described a case of two separate right and left AVs. The right AV drained into the SVC, and the left AV probably drained into the left subclavian vein. Other cases report an AV lying on the midline of the ventral side of VC [14,16], an AV lying on the left side of VC [27], and an AV shifting from the lateral side to the midline of VC and vice versa along its ascending path [21,28].

## 11. Morphological Variations of the AV in Fetuses

Wu et al. [29] established a Z-score formula for the fetal AV and descending aorta based on femur length and gestational age. They studied 452 singleton fetuses with no cardiovascular abnormalities and 25 fetuses with AV-related venous malformations by prenatal sonography. The median gestational age was 24 weeks (19 weeks and 6 days and 40 weeks and 1 day). The diameters of these vessels were measured offline after spatio-temporal image correlation volume acquisition. Using these measurements, the researchers created Z-score formulas for normal AV and descending aorta based on femur length by conducting standard regression analysis, followed by weighted regression of absolute residual values. This study showed a positive linear correlation between AV, femur length, and gestational age. As presented in Table 7, AV Z scores and AV to descending aorta ratios were substantially higher in fetuses with venous malformations than in normal fetuses. 96% of the abnormal group had Z scores greater than +2, and in 96% of cases, the AV to descending aorta ratio was greater than the 95% confidence interval [29].

Krakowiak-Sarnowska et al. [30] studied the variability of the AVS in 32 human fetuses (14 male and 18 female) from the 21st to 24th week of intrauterine life, fixed in 10% neutral formalin solution using conventional anatomical-radiographic methods. Thoracic organs were removed, and the AVS was exposed. The researchers identified five types of AVS. Type 1 (65.6%) had three veins each on the left side of VC, HAV, and AHAV, which joined the AV separately. In the second type (6.25%), the HAV and AHAV joined the AV. In type 3 (12.5%), the AHAV was absent, and the 4th to 8th ICVs joined the AV separately. In the 4th type (6.25%), the HAV was missing, and in the 5th type (9.4%), only the AV was present, passing along the vertebral midline, and the posterior ICVs were also attached to the AV from both sides. In summary, the AV was found in all cases, the HAV in 84.4%, and the AHAV in 80%. An azygos vein system consisting of 3 veins was found in 71.85% of cases, 2 in 18.75%, and 1 in 9.4%. The researchers also examined the AV course. In 90.6% of cases, it was on the right side of the VC. In the rest of the cases, it was in the vertebral midline. In 81.25% of cases, the AV and the SVC connection were projected on T4, 12.5% of cases on T3, and 6.25% on T5. The connection between AV and HAV was found at T8 (35.7%), T9 (18.7%), T10 (17.8%), Th7 (14.2%), and the least frequently at T5, T6, and T11 (3.5%). Between AV and AHAV, it is T7 (41.6%), T6 (29.2%), T8 (25%), and T5 (4.2%).

In another study, Grzybiak et al. [31] compared the occurrence of HAV and AHAV in fetuses, newborns, and adults. They found that HAV was present in 60% of fetuses, 70% of newborns, and 90% of adults, while AHAV was present in only 50% of fetuses and newborns and 56% of adults.

## 12. Clinical Significance

Being aware of the morphological variations of the AV can help clinicians analyze X-rays, CT scans, MRIs, and other imaging studies of the chest and abdomen. There have been cases in the past where abnormal azygos veins have been confused with aneurysms, lymphadenopathies, and tumors [15]. Also, surgeons should be aware of AV abnormalities before performing some thoracic and abdominal surgeries, as each AV type differs in many aspects. Significant changes in the course of the AV, such as the Azygos lobe, should be considered before performing lung surgery.

The azygos lobe is an additional lobe of the right lung that is separated from the superior lobe by an azygos fissure. In the azygos fissure lies the AV, covered by four layers of pleura (in total, two parietal and two visceral layers). The AV and the pleural layers surrounding it are related, like the mesentery and a portion of the small intestine [32]. This variation occurs when, during embryonic development, the right PCV loses its touch with the root of the right lung, which causes it to migrate over the apex of the right lung. As the lungs become bigger, the AV grooves deeper into the lung parenchyma, creating an azygos fissure and an azygos lobe [33,34]. This condition is asymptomatic and does not impact the morbidity of patients [35]. However, it may interrupt thoracoscopic procedures due to possible coexisting abnormalities, such as altered locations of the SVC, right BCV, or esophageal atresia [36,37,38]. Such AV, placed in the azygos fissure, is seen on radiographs as a hair-like strand which stretches between the apex and the root of the right lung [1]. The azygos lobe does not have its own bronchial and blood supply, as it is still a part of the superior lobe of the right lung [39]. The apical and posterior segments of the right superior lobar bronchus divide into the vertical and lateral branches. The vertical branches of both segments are then further divided into the ascending rami, which form the bronchial supply of the azygos lobe, and the descending rami. Venous blood is drained from the azygos lobe through the apical and posterior branches of the right superior pulmonary vein [40]. This obstruction of the superior lobe by the AV can cause clinical symptoms, most often chest pain and dyspnea. The AV can separate from its native azygos fissure and obstruct the right lung in a different place. Such cases can lead to pneumothorax of the right lung, with a visible separation of the parietal and visceral parts of the pleura [41]. It is worth noting that the visceral parts may break apart during the expansion procedure and cause the azygos fissure to disappear. Sporadic cases of a left azygos lobe were documented in the literature, in which the cause of it was a displaced left superior ICV or an improperly retained part of the left PCV, which connected the left SupCV and the left BCV, accordingly to its primitive embryological course [42,43].

Another example of an embryological development error is the absence of the hepatic segment of the IVC with azygos continuation. This phenomenon occurs when, during embryonic development, the right SubCV (the renal part of a mature IVC) fails to connect properly to the hepatic segment of the IVC. In that case, the blood drains into the right SupCV through the right SupSubCa anastomosis. Concurrently, the hepatic veins drain directly into the right atrium. During normal development, the right SupCV loses its connection with the right SupSubCa anastomosis; however, when the hepato-subcardinal connection is not present, the pressure of the blood prevents the constriction of the connection point between the right supracardinal vein and the right SupSubCa anastomosis, causing it to stay patent. Compared to a normal AV, the AV that serves as a continuity of the IVC is significantly larger in diameter [5,44].

The etiology of the AV is, in most cases, correlated with the presence of an “aortic nipple”. The “aortic nipple” is a specific anomaly seen on radiographs at the level of the aortic arch. This term was first used by McDonald et al. [45]. When the AV fails to develop, the blood pressure in the entire AVS rises, leading to the dilation of the veins it comprises and its tributaries. Dilation of the left superior ICV, which usually drains into the SVC and the AHAV, leads to a visible “aortic nipple” on X-ray images at the level of crossing of these two structures [46]. This slight anomaly can help identify a pneumomediastinum or a medial left pneumothorax. In the case of a pneumomediastinum, the medial aspect of the aortic nipple is outlined. In the case of a medial left pneumothorax, the lateral side of the aortic nipple is visibly contoured [47]. Agenesis of the AV can also be correlated with a partial pulmonary anomalous venous return (PPAVR). In such cases, it is essential to consider the altered course of the HAV caused by the agenesis of the AV, as it can be easily mistaken for one of the pulmonary veins [48].

Because of the proximity of the AV and the right bronchus, there is a risk of creating an azygo-tracheal fistula when placing foreign objects, such as a catheter, inside the subclavicular vein or the SVC [49]. On the X-ray image, the tip of the improperly placed catheter will be seen in the azygos arch, or if the catheter lies deeper in the AV, it can be identified as a loop in the right superior mediastinum [50].

Obstruction of the AV, as well as right-sided heart failure, can lead to fluid accumulation in the right costophrenic angle because the AV drains the right parietal pleural veins [51]. Depending on the AVS morphological type, the left pleural veins can be drained by the AV or the HAV, thus making fluid accumulation in the left costophrenic angle possible in case of increased blood pressure in the AVS [2].

The AV arch can significantly increase its diameter. Its physiological mean diameter ranges between 7.8 and 8.2 mm. In some cases, the diameter of the AV arch can reach 60 mm, increasing its size by 7.5 times. This can be caused by reverse blood flow from the SVC or exterior compression, for example, by hilar lymph nodes in chronic inflammatory diseases of the lungs [52].

Aneurysms of the AV are an uncommon and infrequently diagnosed condition of the AV. They can be idiopathic, acquired, or caused by traumatic events. In most cases, they affect the azygos arch, a congenital anatomical weak point. There are no specific symptoms. However, complications may cause rupture, thromboembolism, mediastinal mass effects, and pulmonary hypertension. Treatment is not established; some patients are treated with conservative treatment, including oral anticoagulation. However, surgical treatment like ligation and endovascular occlusion of the aneurysm should be strongly considered in cases with clinical symptoms listed above [53].

Pseudoaneurysms of the AV were also reported in the literature. Nugent et al. [54] published a case report of a 55-year-old woman developing a pseudoaneurysm of the AV after a high-speed motor vehicle collision. However, this condition can also occur spontaneously without any trauma. Usually, it is located at the junction of the trachea and the right upper lobe bronchus. However, in some cases, symptoms such as difficulty breathing, ongoing cough, dysphagia, and a sensation of heaviness in the chest may occur. In severe cases, thoracic pain, particularly in the posterior thorax, respiratory distress, palpitations, and arrhythmia may also be observed. Usually, pseudoaneurysms of the AV are asymptomatic. It is important to note that these pseudoaneurysms and aneurysms of the azygos vein can rupture, causing significant bleeding and hemodynamic instability.

## 13. Conclusions

The AV is morphologically variable in adults and fetuses. The available literature provides numerous morphological types, an understanding of which can facilitate the work of clinicians and surgeons.

## Figures and Tables

**Table 1:** Description of subtypes classified by Kutoglu et al., based on the course of the azygos vein (AV) and its connections with the hemi-azygos vein (HAV) and the accessory hemi-azygos vein (AHAV), their prevalence, and the corresponding types, classified by Anson and McVay, to each subtype.

Number of Subtypes Classified by Kutgolu et al.	Description of Subtypes Described by Kutgolu et al.	Corresponding Type Based on Anson and McVay’s Study	Number of Cases of Each Subtype Found by Kutgolu et al.	Percentage of Cases of Each Subtype Found by Kutgolu et al.
**Group 1**	two separate veins on both sides of the VC with different points of origin	1	1	2.1%
**Group 2**	Both the AV and the HAV had two separate points of origin. The AHAV drained into the HAV, which later drained to the AV. Minor retroaortic anastomoses were present	2	13	27.1%
**Group 3**	The AV and the HAV, with different points of origin, both of these veins, together with AHAV, were connected by a single junction	2	1	2.1%
**Group 4**	The course of all three veins was similar to the ones described in group 2, with a pronounced connection between the AV and the HAV in their proximal parts	2	5	10.4%
**Group 5**	The AV and the HAV had different points of origin and were connected at their caudal parts by a ring-like connection, the AV, HAV, and AHAV formed a ring-like junction at the level of typical drainage point of the HAV into the AV, between both of these ring-like connections, the AV and the HAV were connected by a pronounced anastomosis	2	5	10.4%
**Group 6a**	The AV and the HAV had two different origins. They merged at the halfway point of their course. The AHAV formed and the interazygos vein, and drained into the singular vein formed by the AV and the HAV	2	4	8.3%
**Group 6b**	The course of the veins was similar to that in group 6a; however, the angle of the anastomoses between them was closer to 90^0^ *	2	4	8.3%
**Group 7**	The course of the veins was similar to that in group 6a; however, the interazygos vein was absent	2	11	22.9%
**Group 9**	The AV was the pronounced main vein of the azygos venous system; the HAV was very underdeveloped, compared to the AV	2	1	2.1%
**Group 11**	One main vein, the AV, was present, with multiple short tributaries	3	1	2.1%
**Atypical**	—	–	2	4.2%

AV, azygos vein; HAV, hemiazygos vein; AHAV, accessory hemiazygos vein, *, ^0^ degrees.

**Table 2:** Azygos vein termination level.

Vertebrae Levels	Frequency	Percent
T2–3	6	12.5
T2	6	12.5
T3–4	3	6.3
T3	30	62.5
Missing	3	6.3
Total	48	100

**Table 3:** Sex distribution of the subjects.

Characteristic	Frequency	Percent
Male	61	59.2
Female	42	40.8
Total	103	100

**Table 4:** Subject’s ages and diameter of the azygos vein (AV) (mm).

Characteristic	N	Minimum	Maximum	Mean
Age	103	18.0	81.00	47.0818
AV diameter	103	4.30	16.00	8.1126
Males	61	4.70	16.00	8.2
Females	42	4.30	15.55	7.8

**Table 5:** The diameter of the azygos vein (AV) at the origin and termination levels.

Characteristic	N	Range	Minimum	Maximum	Mean
Age	48	43	27	70	48.29
DAVO	48	5.5	2.0	7.5	4.050
DAVT	45	7.2	5.0	12.2	8.558

DAVO, Diameter of azygos vein (origin); DAVT, Diameter of azygos vein (termination).

**Table 6:** Distribution of the azygos vein horizontal positions at five vertical levels.

Vertical Level	RM	RL	RP	M	LP	LL	LM	Modal Position
(5) Superiormost (~T4)	8	13	19	23	13	6	1	4
(4) Superior Intermediate	4	7	10	16	27	17	2	5
(3) Middle	4	6	15	16	20	15	7	5
(2) Inferior Intermediate	5	10	17	24	19	7	1	4
(1) Inferiormost (~T12)	12	24	28	11	7	-	1	3

RM, rightmost; RL, right lateral; RP, right paramedian; M, median; LP, left paramedian; LL, left lateral; and LM, leftmost.

**Table 7:** Comparison of cardiovascular parameters between abnormal and normal groups.

Characteristic	Abnormal Group (n = 25)	Normal Group (n = 452)
AV Z score	4.38 (1.51)	−0.14 (1.35)
DAo Z score	0.47 (1.64)	−0.05 (1.32)
AV/DAo ratio	0.70 (0.08)	0.38 (0.11)

AV, azygos vein; DAo, descending aorta.
